# Tangshen Formula Attenuates Diabetic Kidney Injury by Imparting Anti-pyroptotic Effects *via* the TXNIP-NLRP3-GSDMD Axis

**DOI:** 10.3389/fphar.2020.623489

**Published:** 2021-01-29

**Authors:** Nan Li, Tingting Zhao, Yongtong Cao, Haojun Zhang, Liang Peng, Yan Wang, Xuefeng Zhou, Qian Wang, Jialin Li, Meihua Yan, Xi Dong, Hailing Zhao, Ping Li

**Affiliations:** ^1^ Graduate School of Peking Union Medical College, Chinese Academy of Medical Science & Peking Union Medical College, Beijing, China; ^2^ Beijing Key Laboratory for Immune-Mediated Inflammatory Diseases, Institute of Clinical Medical Sciences, China-Japan Friendship Hospital, Beijing, China; ^3^ Clinical Laboratory, China-Japan Friendship Hospital, Beijing, China

**Keywords:** tangshen formula, diabetic kidney disease, NLRP3 inflammasome, pyroptosis, reactive oxygen species, thioredoxin interacting protein

## Abstract

We previously reported that Tangshen formula (TSF), a Chinese herbal medicine for diabetic kidney disease (DKD) therapy, imparts renal protective effects. However, the underlying mechanisms of these effects remain unclear. Pyroptosis is a form of programmed cell death that can be triggered by the NLRP3 inflammasome. Recently, the association between the pyroptosis of renal resident cells and DKD was established, but with limited evidence. This study aimed to investigate whether the renal protective effects of TSF are related to its anti-pyroptotic effect. DKD rats established by right uninephrectomy plus a single intraperitoneal injection of STZ and HK-2 cells stimulated by AGEs were used. *In vivo*, TSF reduced the 24 h urine protein (24 h UP) of DKD rats and alleviated renal pathological changes. Meanwhile, the increased expression of pyroptotic executor (GSDMD) and NLRP3 inflammasome pathway molecules (NLRP3, caspase-1, and IL-1β) located in the tubules of DKD rats were downregulated with TSF treatment. *In vitro*, we confirmed the existence of pyroptosis in AGE-stimulated HK-2 cells and the activation of the NLRP3 inflammasome. TSF reduced pyroptosis and NLRP3 inflammasome activation in a dosage-dependent manner. Then, we used nigericin to determine that TSF imparts anti-pyroptotic effects by inhibiting the NLRP3 inflammasome. Finally, we found that TSF reduces reactive oxygen species (ROS) production and thioredoxin-interacting protein (TXNIP) expression in AGE-stimulated HK-2 cells. More importantly, TSF decreased the colocalization of TXNIP and NLRP3, indicating that ROS-TXNIP may be the target of TSF. In summary, the anti-pyroptotic effect *via* the TXNIP-NLRP3-GSDMD axis may be an important mechanism of TSF for DKD therapy.

## Introduction

Diabetic kidney disease (DKD) is a severe microvascular complication of diabetes and is also the leading cause of end-stage renal disease (Umanath and Lewis 2018). The pathogenesis of DKD is complex and mainly involves hemodynamic disorders, oxidative stress, chronic-inflammatory injuries, overactivation of the renin-angiotensin-aldosterone system (RAAS), and dyslipidemia (Lin et al., 2018; Matoba et al., 2019). Recently, the pyroptosis of renal resident cells has been associated with DKD. However, evidence remains limited (Li et al., 2017; Wang et al., 2019; Gu et al., 2019b).

Pyroptosis is a type of programmed cell death that has recently been reported to occur in various diseases (Wang et al., 2019; Wu et al., 2019; Zhaolin et al., 2019; Yu et al., 2020a). In the canonical pyroptotic pathway, pyroptosis is dependent on cleaved caspase-1, whereas in the non-canonical pathway, caspase-4/5/11 mediates pyroptosis (Shi et al., 2017). The membrane protein gasdermin D (GSDMD) is a member of the gasdermin (GSDM) family, which has been shown to be the executor of pyroptosis (Aglietti and Dueber 2017; Feng et al., 2018). GSDMD can be cleaved by activated caspases into the N-and C-termini. The N-terminus of GSDMD (GSDMD-N) has pore-forming activity, which destroys membrane integrity and triggers the release of cellular contents and proinflammatory cytokines. Thus, pyroptosis is also considered as a type of inflammatory cell death (Broz et al. 2020).

Inflammasomes trigger pyroptosis (Lamkanfi and Dixit, 2014; Xue et al., 2019) by inducing the generation of cleaved caspase-1 during inflammasome activation. Among known inflammasomes, the NLRP3 inflammasome is the most extensively studied. It is a multi-protein complex located in the cytoplasm and is composed of pattern recognition receptor NLRP3, apoptosis-associated speck-like protein (ASC), and caspase-1 (Martinon et al., 2002). It is assembled and activated by a variety of signals, which lead to the self-cleavage of the precursor form of caspase-1 (pro-caspase-1) into its activated form (cleaved caspase-1). The activated caspase-1 is the functional unit of the NLRP3 inflammasome that cleaves the precursor of IL-1 β and IL-18 (pro-IL-1 β and pro-IL-18) into the mature forms (mIL-1β and mIL-18), which are then secreted and impart inflammatory effects. Accumulating evidence shows that the chronic inflammation caused by the NLRP3 inflammasome is related to DKD (Shahzad et al., 2015; Wada and Makino 2016; Wu et al., 2018). However, the evidence for NLRP3-related pyroptosis in DKD is limited.

Oxidative stress is the main contributor to DKD. Overproduction of reactive oxygen species (ROS) has been proven to be an important factor in NLRP3 inflammasome activation (Ding et al., 2018). Thioredoxin-interacting protein (TXNIP) is a cellular regulator of oxidative stress that inhibits the antioxidant activity of thioredoxin (TRX) and exacerbates oxidative stress (Li et al., 2014a; Yoshihara et al., 2014). In addition, TXNIP has been reported to increase inflammation by activating the NLRP3 inflammasome, leading to renal injuries in DKD (Han et al., 2018). However, the involvement of ROS-TXNIP in NLRP3-related pyroptosis in DKD remains unclear.

Tangshen formula (TSF) is a traditional Chinese medicine for the treatment of DKD. In our previous clinical trials, we showed that TSF decreases macro-proteinuria in stage IV DKD patients, increases estimated glomerular filtration rate (eGFR), and improves dyslipidemia and abdominal circumference (Li et al., 2015). *In vivo* and *in vitro* experiments have demonstrated that TSF plays renal protective effects *via* reducing inflammation and fibrosis, regulating cholesterol metabolism, and promoting autophagy (Zhao et al., 2016; Zhao et al., 2017; Liu et al., 2018a). This study aimed to explore the potential anti-pyroptotic effect of TSF *via* the TXNIP-NLRP3-GSDMD axis.

## Materials and Methods

### Herbal Formulation and Components

Seven herbs constitute TSF: 35.3% astragalus root (Astragali radix), 17.6% burning bush twig (Euonymi ramulus), 14.4% rehmannia root (Rehmanniae radix), 11.5% bitter orange (Aurantii fructus), 10.6% cornus fruit (Corni fructus), 7.1% rhubarb root and rhizome (Rhei radix et rhizoma), and 3.5% notoginseng root (Notoginseng radix). The TSF powder used in this study was prepared and standardized by an established company (Jiangyin Tianjiang Pharmaceutical, Jiangsu, China; http://www.tianjiang.com) according to the guidelines in the Pharmacopoeia of The People’s Republic of China 2010. The major chemical compositions (loganin, calycosin-7-O-b-D-glucoside, naringenine-7-rhamnosidoglucoside, neohesperidin, naringenin, and aloeemodin) of TSF were verified in our previous study (Kong et al., 2016).

### Animal Experimental Design and Drug Administration

Six-week-old Sprague Dawley (SD) rats weighing 135–160 g were purchased from Beijing HFK Bio-Technology Co. Ltd. (China). The DKD model was established as previously described (Zhao et al., 2012). Briefly, the SD rats underwent right uninephrectomy to accelerate DKD. Seven days after surgery, STZ (Sigma, USA), which was diluted in citrate buffer (0.1 mol/L, pH 4.5), was intraperitoneally injected into the rats at a dose of 40 mg/kg. Three days after STZ injection, blood glucose levels were assessed to confirm the diabetic state. Rats with blood glucose levels >16.7 mmol/L were qualified as diabetic and randomly assigned to the vehicle-treated or TSF-treated group. Control rats received a sham operation involving laparotomy and manipulation of renal pedicles. Seven days later, an intraperitoneal injection of citrate buffer (0.1 mol/L, pH 4.5) was performed on the sham-operated rats. Chloral hydrate at a dosage of 330 mg/kg was injected prior to surgery.

**FIGURE 1:**
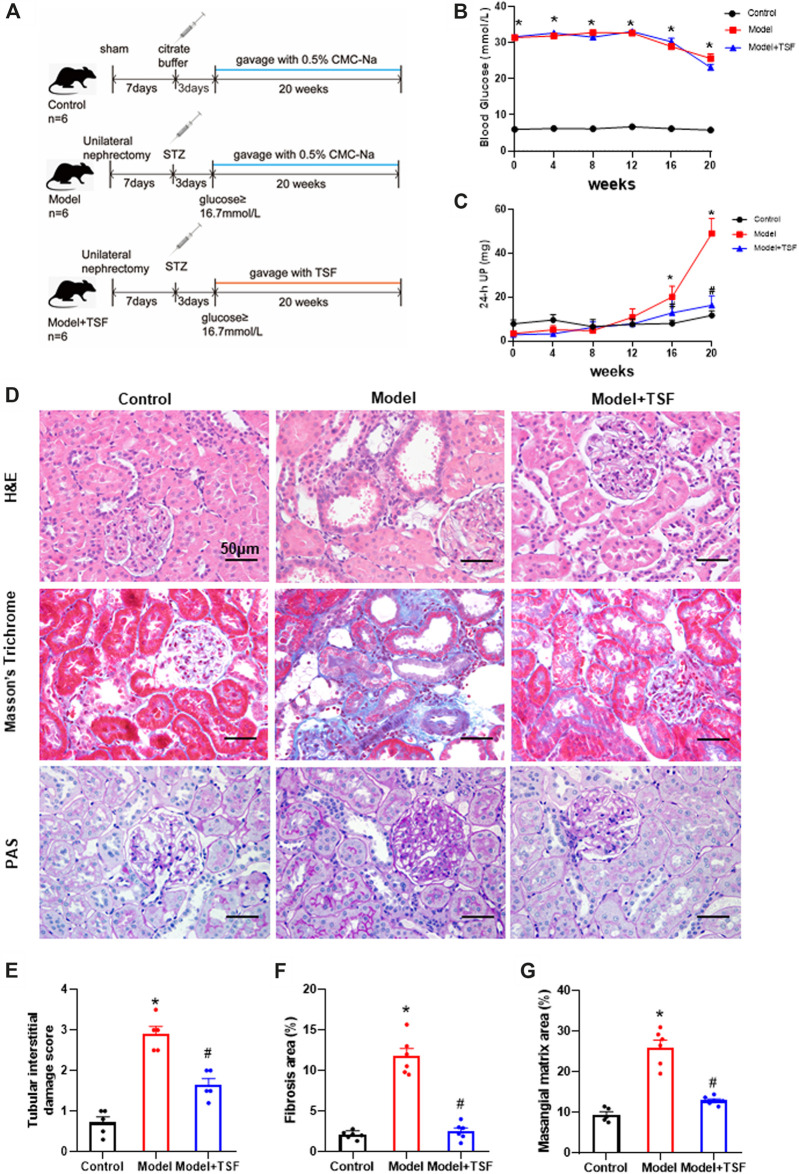
TSF alleviates proteinuria and pathological kidney injuries in DKD rats. **(A)** Animal experiment protocol. **(B and C)** Blood glucose and 24 h UP were detected every four weeks. **(D)** H&E, Masson’s trichrome, and PAS staining were performed to evaluate kidney injury. **(E)** Tubular damage scores based on H&E staining. **(F)** Semi-quantification of collagen areas according to Masson’s trichrome staining. **(G)** Semi-quantification of mesangial matrix area based on PAS staining. The magnification of the images is ×400. The data are expressed as the mean ± SEM. * indicates *p* < 0.05 *vs*. Control; # indicates *p* < 0.05 vs. Model.

The following groups were included in this study: 1) Control group (n = 6) received 0.5% CMC-Na solution, 2) Model group (n = 6) received 0.5% CMC-Na solution, and 3) Model + TSF group (n = 6) received TSF treatment. Treatment was initiated on the third day after citrate buffer/STZ injection and continued for 20 weeks. To ensure the accuracy of administration, TSF was suspended in 0.5% CMC-Na solution, which helps the drug to be dispersed evenly. The treating dosage for rats was 2.4 g/kg body weight/day, which was six times greater than the clinical dosage (0.4 g/kg body weight/day) (Yan et al., 2016). Blood glucose and 24 h urine volume were measured every four weeks. At the end of the experiment, serum and renal tissues were collected for follow-up assessment. The animal experiment design is presented in Figure 1A.

All procedures were approved by the Ethics Committee of China-Japan Friendship. Hospital, Institute of Clinical Medical Science (No. 2012-A04) and performed in accordance with the National Academies Guiding Principles for the Care and Use of Laboratory Animals, 8th edition.

### Measurement of Blood Glucose and Urine Protein

During the experiment, glucose was monitored using One Touch Ultra II system (LifeScan, USA) every four weeks using tail vein blood. The 24 h urine protein (24 h UP) was measured by an AU5800 automatic biochemistry analyzer (Beckman, USA).

### Histological Examination

Paraffin-embedded kidney sections were examined for pathological injuries. Tubulointerstitial damage was assessed by H&E staining based on the established scoring system (Zhao et al., 2016). Interstitial fibrosis was evaluated using Masson’s trichrome stain. Periodic acid-Schiff (PAS) staining was performed to evaluate extracellular matrix (ECM) deposition in the mesangial area. Semi-quantitative analysis was performed using Image-Pro Plus 6.0.

### Immunohistochemistry Staining of GSDMD, NLRP3, and IL-1β

The kidney sections were incubated with anti-GSDMD (1:50, Proteintech, China), anti-NLRP3 (1:200, Proteintech), or anti-IL-1β (1:500, Abcam) after microwave-based antigen retrieval. Positive staining patterns were identified under ×400 magnification, and 10 fields were randomly selected for semi-quantitative analysis. Integrated option density (IOD) values in the examined field were measured using Image-Pro Plus 6.0.

### Immunofluorescence Assay

Paraffin-embedded kidney sections were used to assess colocalization of caspase-1 and GSDMD or NLRP3. First, sections were subjected to microwave-based antigen retrieval. Then, tyramide signal amplification (TSA) was performed as previously described (Faget and Hnasko 2015). Briefly, the following steps were performed: 1) incubating sections with anti-NLRP3 (1:1,000, Proteintech) or anti-GSDMD (1:500, Proteintech) at 4°C overnight, 2) incubating with horseradish peroxidase (HRP)-conjugated secondary antibody for 50 min at room temperature, 3) reacting with CY3-TSA (Servicebio, China) for 10 min in the dark, 4) removing nonspecific binding antibodies by microwave treatment, 5) incubating with anti-caspase-1 (1:50, Proteintech) at 4°C overnight, 6) incubating with Alexa Fluor 488-conjugated goat anti-rabbit IgG (Servicebio, China) for 50 min in the dark, and 7) staining with DAPI solution for 10 min. Images were captured by fluorescence microscopy using excitation wavelengths of 510–560 nm (red) and 465–495 nm (green).

### Cell Culture and Intervention

The human proximal tubular epithelial cell line (HK-2) was obtained from the National Infrastructure of Cell Line Resource (China). The HK-2 cells were cultured in RPMI-1640 medium (Gibco) containing 10% fetal bovine serum at 37°C and 5% CO_2_ air. To induce cell death, AGE-BSA (200 μg/ml, Abcam) was used to stimulate HK-2 cells for 48 h. AC-yvad-cmk (10 μg/ml, Sigma) was used in the pretreatment of cells for 0.5 h before AGE stimulation to confirm pyroptosis of HK-2 cells. The activation of the NLRP3 inflammasome in HK-2 cells treated by AGEs for different lengths of time (12 h, 24 h, 48 h) was also assessed. According to the CCK8 assay (Supplementary Figure S1) and our previous study (Liu et al., 2018b), TSF of 250 μg/ml or 500 μg/ml was used to co-treat cells with AGEs. To explore the anti-pyroptotic mechanism of TSF, nigericin (10 μM, InvivoGen, USA) was added to the AGE and TSF co-treated cells 1 h before harvesting the cells.

### Calcein-AM/PI Double Stains

A calcein-AM/PI double staining kit was purchased from Dojindo Laboratories. The fluorescent dye Hoechst 33342 was obtained from Sigma. First, a calcein-AM/PI/Hoechst working solution was prepared using 2 μM calcein-AM, 4.5 μM PI, and 3.6 μM Hoechst 33342. After intervention, the HK-2 cells of each group were washed with PBS once, then stained with 100 μL calcein-AM/PI/Hoechst working solution and incubated at 37°C for 15 min. After incubation, the cells were washed twice. The cells were assessed under a fluorescence microscope (×200) or detected by flow cytometry to determine the percentage of dead/live cells.

### Western Blot Analysis

Protein samples extracted from renal tissues or HK-2 cells were used. SDS-PAGE and PVDF membrane transfer were performed as previously described (Wang et al., 2019). After blocking for 1 h at room temperature, the following primary antibodies were used: anti-GSDMD-N (1:1000, CST, USA), anti-cleaved caspase-1 (1:1000, CST), anti-NLRP3 (1:1000, Proteintech), anti-IL-1β (1:1000, Abcam), anti-IL-18 (1:1000, Proteintech), anti-TXNIP (1:1000, Abcam), and anti-β-actin (1:1000, Santa Cruz, USA). Secondary antibodies were used at a dilution of 1:5000. Signals were detected with a ChemiDoc XRS system (Bio-Rad, CA, USA). ImageJ software (NIH, Bethesda, MD, USA) was used to quantify the protein bands against *β*-actin.

### Assessment of IL-1β Release by ELISA

IL-1β levels in the cell supernatant were determined using a human IL-1β ELISA kit (CUSABIO, China) according to the manufacturer’s instructions.

### ROS Production

To detect ROS production, the HK-2 cells from different treatment groups were incubated with dihydroethidium (DHE) dye (Beyotime, China) for 30 min at a concentration of 5 μM. Hoechst 33342 (Sigma) at a concentration of 3.6 μM was added to staining nuclei. The results were observed under a fluorescence microscope (×200) at an excitation wavelength of 535 nm.

### Cell Immunofluorescence

HK-2 cells were fixed with 4% paraformaldehyde for 20 min after treatment, then blocked with BSA for 30 min at room temperature. The fixed cells were incubated with a primary antibody cocktail against NLRP3 (1:100, Proteintech) and TXNIP (1:100, Abcam) at 4°C overnight. After washing twice with PBS, the cells were incubated with CoraLite and Rhodamine-conjugated secondary antibody solution (1:100, Proteintech) at 37°C for 1 h. The cells were washed twice with PBS and then stained with DAPI (Solarbio, China) to detect nuclei. Cell images were captured using a confocal laser scanning microscope at ×900 magnification. Laser excitation wavelengths of 570 nm and 488 nm were used to observe red and green fluorescence.

### Statistical Analysis

The GraphPad Prism software version 8.0 was used for analysis. Quantitative data were expressed as the mean ± SEM. One-way ANOVA was used to compare groups. Differences with *p* < 0.05 were considered statistically significant.

## Results

### TSF Attenuates the Kidney Injuries *in Vivo*

During the experiment, the blood glucose levels of DKD rats remained at a relatively high level (≥23.1 ± 0.85 mmol/L). TSF treatment had no effects on blood glucose levels. The 24 h UP level of the DKD rats continuously increased and was significantly higher than the control from the 16th week of this experiment. However, in the TSF-treated rats, the 24 h UP did not markedly increase (Figures 1B,C).

H&E, Masson’s trichrome, and PAS staining were performed to evaluate renal pathological injury (Figures 1D–G). In the DKD rats, tubular epithelial cell swelling, detachment, and vacuolar degeneration, as well as obvious inflammatory cell infiltration were observed with H&E staining. Furthermore, cast and cell debris were detected in some dilated tubular lumens. These changes were significantly alleviated in TSF-treated DKD rats (Figures 1D,E). Masson’s trichrome stain and semi-quantitative analysis showed enhanced tubulointerstitial fibrosis in DKD rats compared with the control, which was attenuated in TSF-treated rats (Figures 1D,F). PAS staining and semi-quantitative analysis showed that glomerular mesangial matrix expansion was enhanced in the DKD rats compared with the control; however, this enhancement was attenuated in DKD rats treated with TSF (Figures 1D,G).

### TSF Downregulates Expression of GSDMD, NLRP3, IL-1β, IL-18, and Caspase-1 *in vivo*

**FIGURE 2:**
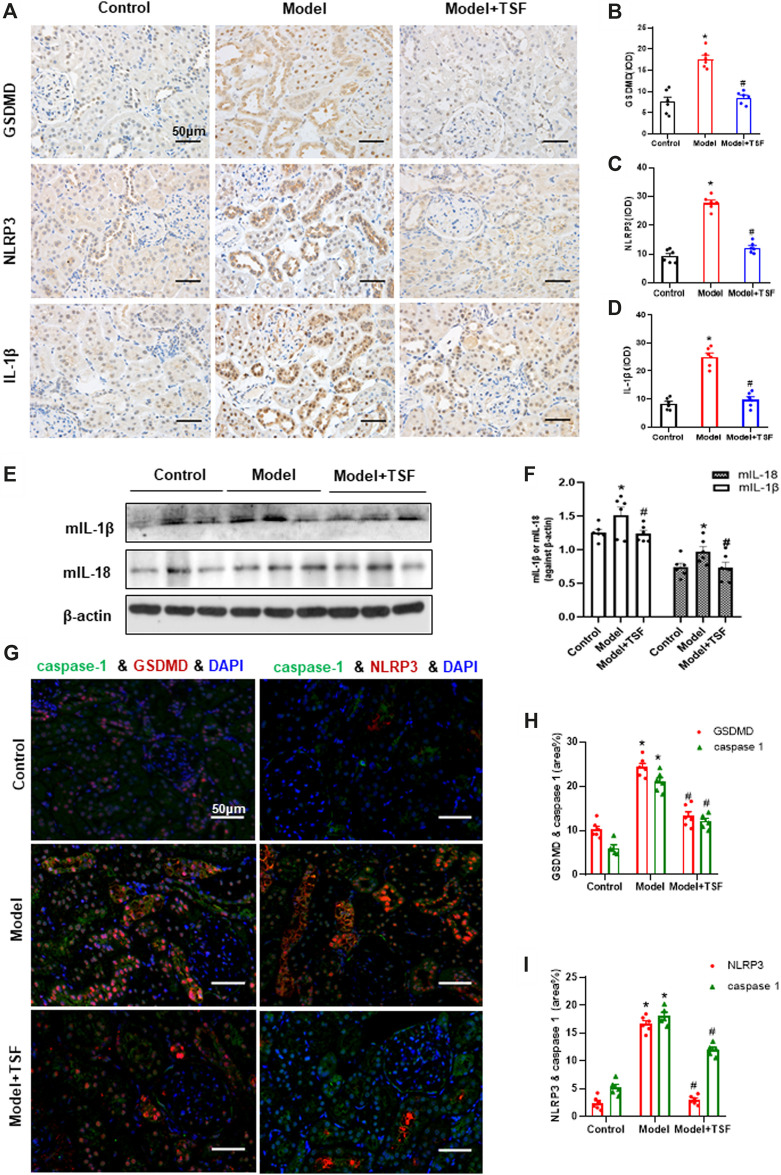
TSF downregulates the renal expression of GSDMD (pyroptosis executor) and NLRP3 inflammasome in DKD rats. **(A–D)** Expression levels of GSDMD, NLRP3, and IL-1β in the kidney were detected by IHC and analyzed semi-quantitatively. **(E, F)** Expression levels of mIL-1β and mIL-18 were detected by WB assay and analyzed semi-quantitatively. **(G)** The colocalization of caspase 1 with GSDMD or NLRP3 in the kidney is shown by immunofluorescence. The magnification of the images is ×400. **(H, I)** Positive area (%) of fluorescent staining was analyzed by ImageJ software. Semi-quantitative values were expressed as the mean ± SEM. * indicates *p* < 0.05 vs. Control; # indicates *p* < 0.05 *vs*. Model.

Proteins in the pyroptotic pathway were detected in the kidney. IHC and semi-quantitative analyses showed that the expression of GSDMD, NLRP3, and IL-1β was much higher in DKD rats than the controls (Figure 2A–D). In addition, western blot assay showed that the expression of mIL-1β and mIL-18 was also higher in DKD rats than the controls (Figures 2E,F). However, in TSF-treated rats, the expression of the above molecules was significantly downregulated (Figures 2A–F). Caspase-1 is involved in the canonical pyroptotic pathway. Therefore, in this experiment, immunofluorescence was performed to co-localize caspase-1 with GSDMD or NLRP3 in the kidney. Figures 2G–I show that caspase-1 co-localized with GSDMD or NLRP3 mainly in the tubules, and colocalization was significantly higher in DKD rats compared with the controls. However, in TSF-treated rats, colocalization of pyroptotic-related proteins was effectively inhibited. Original pictures of Figure 2G were uploaded to the Supplementary Materials.

### TSF Reduces AGE-Induced Pyroptosis *in vitro*

**FIGURE 3:**
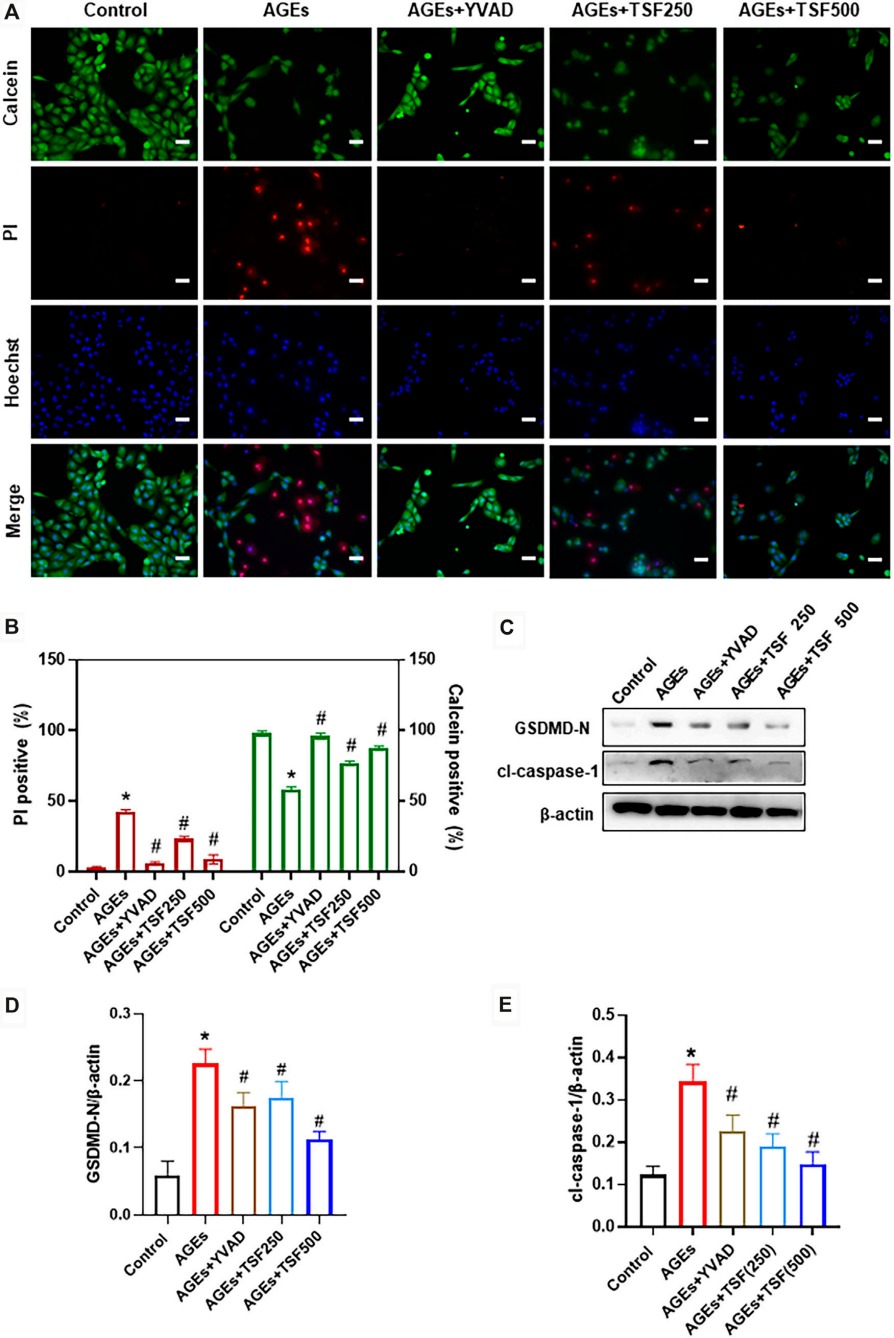
TSF decreases AGE-induced pyroptosis of HK-2 cells. Cell death was induced by AGE (200 μg/ml) stimulation for 48 h. **(A)** Representative pictures of PI/calcein-AM double stains. PI-positive cells indicate dead cells, and live cells were stained by calcein. Ten fields (×200) were randomly selected to calculate the percentage of dead/live cells. To prove that AGE-induced cell death is caspase-1-dependent, YVAD (caspase-1 inhibitor) was used to pretreat cells before AGE stimulation. In this part of the experiment, 250 μg/ml and 500 μg/ml of TSF were used to co-treat HK-2 cells with AGEs. **(B)** The ratio of positive cells was calculated according to the *p*I/Calcein-AM double stains. **(C–E)** Relative expression levels of GSDMD N-terminus and cleaved caspase-1 were assessed by western blotting. Scale bar = 100 μm. Data were expressed as the mean ± SEM. * indicates *p* < 0.05 *vs*. Control; # indicates *p* < 0.05 *vs*. AGEs.

To explore the anti-pyroptotic effect of TSF, AGE-stimulated HK-2 cells were assessed. Calcein-AM/PI double staining was performed to identify live/dead cells. With AGE stimulation for 48 h, the percentage of calcein-positive cells significantly decreased, whereas that of PI-positive cells significantly increased (Figures 3A,B). To confirm that AGE-induced cell death is caspase-1-dependent, the HK-2 cells were pretreated with YVAD (caspase-1 inhibitor) before AGE stimulation. Calcein-AM/PI double staining showed that the number of dead cells significantly decreased with YVAD pre-treating, indicating that AGE-induced cell death is mainly caspase-1-dependent. Accordingly, GSDMD N-terminus (GSDMD-N) and cleaved-caspase-1 (cl-caspase-1) were highly expressed in AGE-stimulated HK-2 cells, whereas with YVAD pretreatment they were significantly downregulated (Figures 3C–E). These results support the existence of pyroptosis in AGE-stimulated HK-2 cells. TSF (250 μg/ml, 500 μg/ml) imparted anti-pyroptotic effects, reducing the number of pyroptotic cells as well as the expression of GSDMD-N and cl-caspase-1 in a dosage-dependent manner (Figure 3).

### TSF Inhibits the AGE-Induced NLRP3 Inflammasome Activation *in vitro*

**FIGURE 4:**
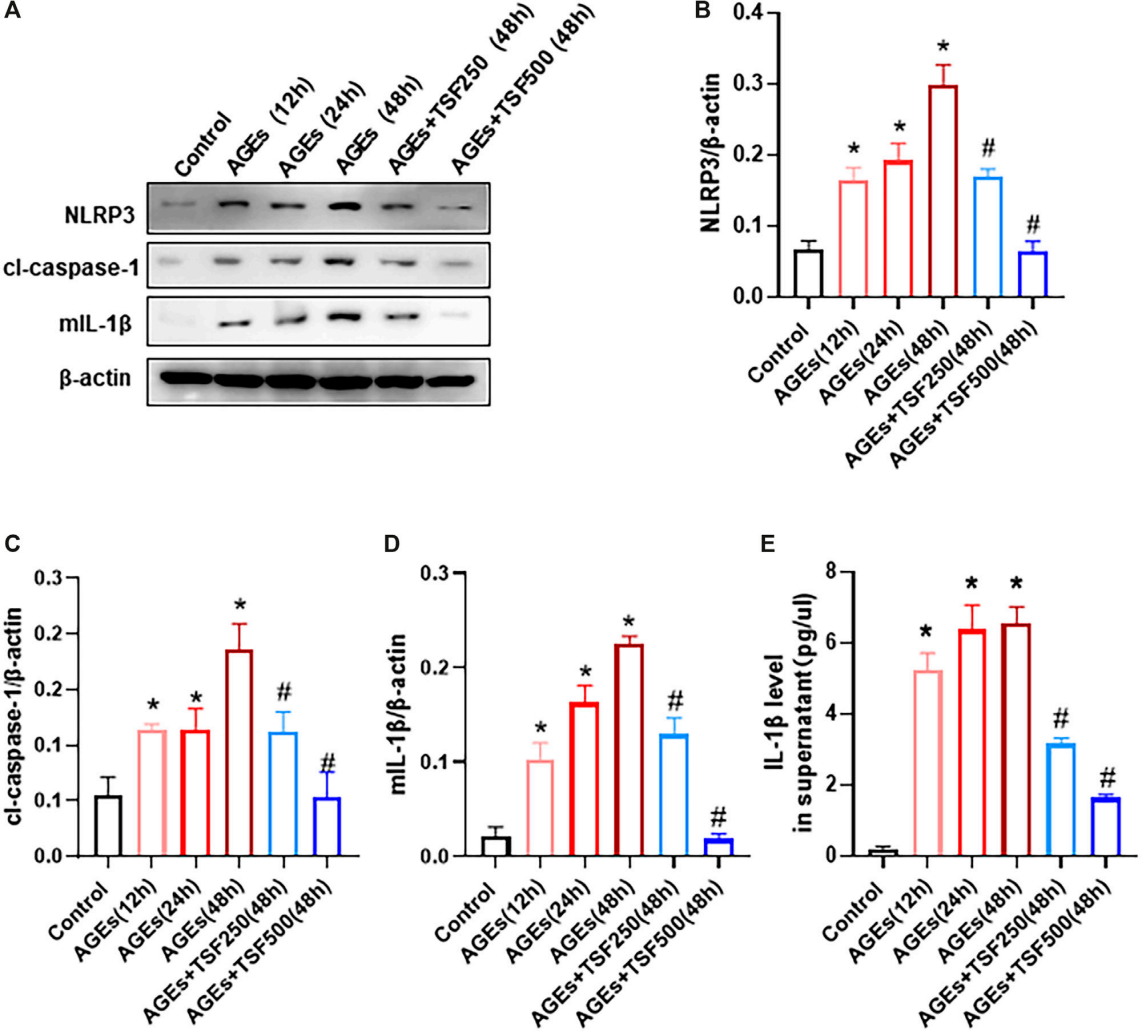
TSF inhibits the activation of the NLRP3 inflammasome in AGE-stimulated HK-2 cells. AGE-stimulated HK-2 cells were assessed at three time points (12 h, 24 h, 48 h). TSF at concentrations of 250 μg/ml and 500 μg/ml was used to co-treat HK-2 cells with AGEs for 48 h **(A–D)** Expression levels of NLRP3, cleaved-caspase 1, and IL-1β were assessed by western blotting. **(E)** IL-1β levels in the supernatant were determined by ELISA. The data were expressed as the mean ± SEM. * indicates *p* < 0.05 vs. Control; # indicates *p* < 0.05 *vs*. AGEs (48 h).

To detect activation of the NLRP3 inflammasome as a trigger of pyroptosis, the expression of NLRP3, cl-caspase-1, and mature IL-1β (mIL-1β) in AGE-stimulated HK-2 cells was assessed and was shown to increase in a time-dependent manner (Figure 4A–D). In addition, IL-1β levels in the supernatant of HK-2 cells increased (Figure 4E), indicating the activation of the NLRP3 inflammasome. However, TSF (250 μg/ml, 500 μg/ml) treatment decreased the expression of NLRP3, cl-caspase-1, and mIL-1β, as well as released IL-1β in a dose-dependent manner (Figure 4).

### Anti-Pyroptotic Effect of TSF is Abolished by NLRP3 Inflammasome Agonist *in vitro*

**FIGURE 5:**
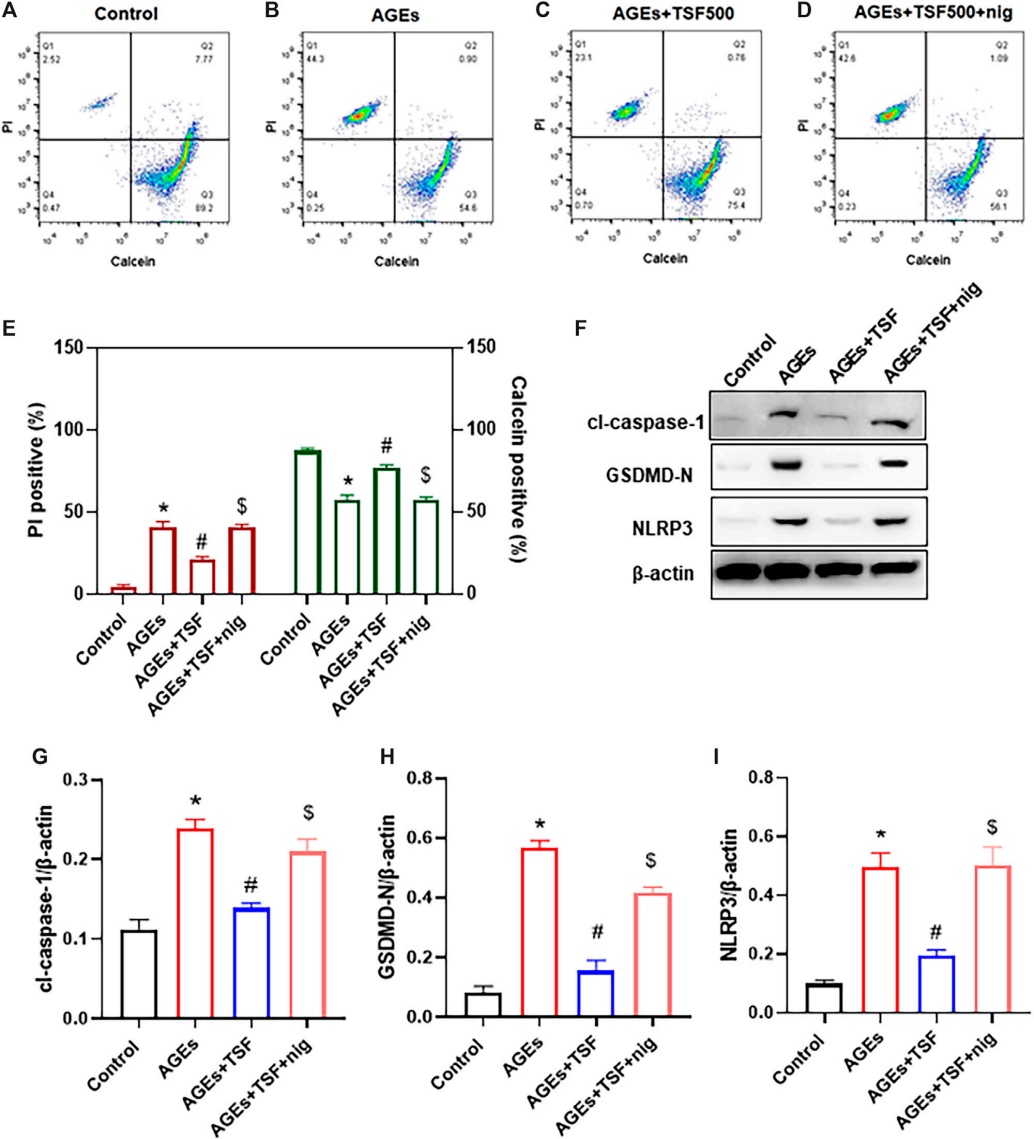
TSF decreases pyroptosis by inhibiting the activation of the NLRP3 inflammasome in AGE-stimulated HK-2 cells. HK-2 cells were treated with TSF of 500 μg/ml **(A–E)** HK-2 cells stained with PI/calcein-AM in each treated group were detected by flow cytometry, and the ratios of PI-positive or calcein-positive cells were calculated. Nigericin (NLRP3 inflammasome agonist) was added to AGE-and TSF-co-treated cells 1 h before cell collection. **(F–I)** Relative expression of GSDMD-N, NLRP3, and cleaved-caspase-1 was detected by western blotting. The data were expressed as the mean ± SEM. “nig” indicates nigericin; * indicates *p* < 0.05 vs. Control; # indicates *p* < 0.05 *vs*. AGEs; $ indicates *p* < 0.05 *vs*. AGEs + TSF.

To confirm that TSF affects pyroptosis by regulating the NLRP3 inflammasome, the exogenous agonist of NLRP3 inflammasome nigericin was employed in this experiment together with the TSF at a concentration of 500 μg/ml. Pyroptotic cells were detected by calcein-AM/PI double staining, and the percentage of positive cells was determined by flow cytometry. Similar to the results of the previous experiment, flow cytometry also showed that TSF decreased the ratio of pyroptotic cells induced by AGEs (Figures 5A–C, E). Furthermore, the addition of nigericin to cells co-treated with AGEs and TSF for 1 h before harvest resulted in the restoration of the ratio of pyroptotic cells (Figures 5D,E). In addition, nigericin restored the protein expression of GSDMD-N, cl-caspase-1, and NLRP3, which were inhibited by TSF treatment (Figures 5F–I). These results suggest that TSF decreases pyroptosis mainly by inhibiting the activation of the NLRP3 inflammasome.

### TSF Reduces ROS Production and TXNIP-NLRP3 Interactions *in vitro*

**FIGURE 6:**
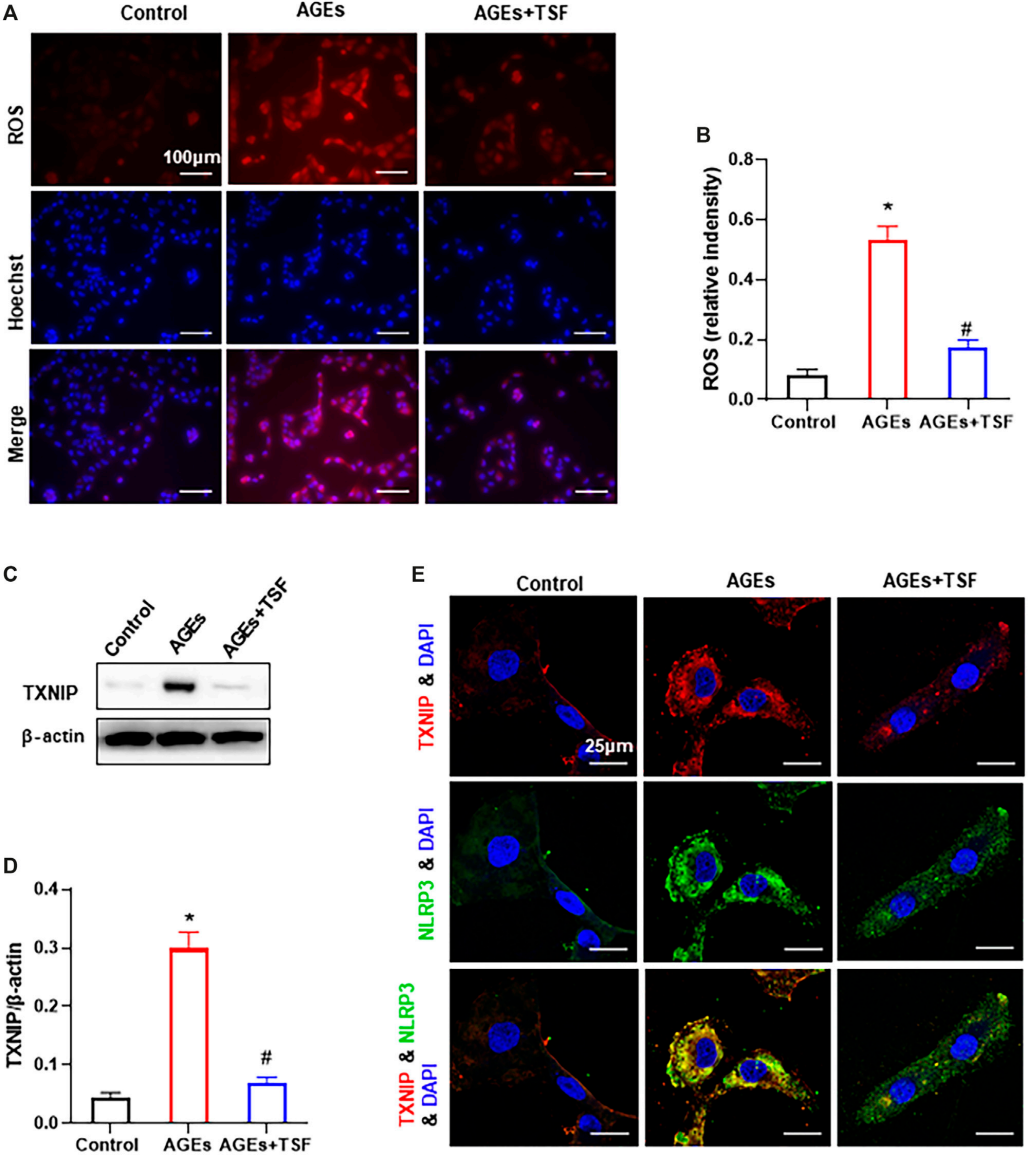
TSF reduces ROS production and TXNIP-NLRP3 colocalization in AGE-stimulated HK-2 cells. TSF at a concentration of 500 μg/ml was used in the experiment. **(A)** ROS production was tested by a DHE probe (magnification: 200×, scale bar = 100 μm). **(B)** Relative fluorescence intensity was calculated by DHE against Hoechst. **(C and D)** Expression of TXNIP was assessed by western blotting. **(E)** Colocalization of TXNIP and NLRP3 is shown by immunofluorescence (magnification: ×900, scale bar = 25 μm). The data are presented as the mean ± SEM. * indicates *p* < 0.05 vs. Control; # indicates *p* < 0.05 *vs*. AGEs.

The ROS-TXNIP pathway has been shown to activate the NLRP3 inflammasome in DKD. In this experiment, TSF (500 μg/ml) was used to treat AGE-stimulated HK-2 cells. DHE probe analysis showed an increase in ROS production in AGE-stimulated HK-2 cells (Figures 6A,B). As a regulator of cellular oxidative stress, the expression of TXNIP was significantly upregulated in AGE-stimulated cells compared with the control (Figures 6C,D). In addition, the colocalization of TXNIP with NLRP3 also increased (Figure 6E). However, with TSF treatment, ROS production, TXNIP expression, and TXNIP-NLRP3 colocalization decreased, which suggests that ROS-TXNIP is the target of TSF.

## Discussion

The present study provides evidence that the Chinese herbal medicine TSF significantly reduces urine protein levels and alleviates diabetic kidney injuries in DKD rats. These changes might be related to the anti-pyroptotic effect of TSF. In detail, TSF protected tubular epithelium from pyroptosis by regulating the TXNIP-NLRP3-GSDMD axis.

The pathogenesis of DKD is complex and involves multiple pathways. In our previous studies, TSF was found to affect multiple signaling pathways. It has been reported to impart anti-inflammation and anti-fibrotic effects in diabetic rats by interfering with the NF-κB and TGF-β/Smad3 signaling pathways (Zhao et al., 2016). TSF has also been found to increase cellular autophagy by inhibiting PLZF expression in db/db mice (Zhao et al., 2017). Recently, also in db/db mice, TSF was shown to promote ABCA1-mediated renal cholesterol efflux, which contributes to its therapeutic effects (Liu et al., 2018a). In the present study, we show that the pharmacological mechanism of TSF in alleviating diabetic kidney injuries involves imparting anti-pyroptotic effects.

Pyroptosis is a form of programmed cell death that has been investigated in the past few years (Shi et al., 2017; Yu et al., 2020b). It differs from apoptosis in that the canonical pyroptotic pathway is dependent on activated caspase-1, which in turn hydrolyzes the membrane protein GSDMD at the N-and C-termini. The N-terminus of GSDMD has pore-forming activity, which oligomerizes to membranes to drill pores. To disrupt membrane integrity, pyroptosis occurs with cell swelling and the release of intracellular contents, which involves the inflammatory cytokines IL-1β and IL-18. These findings indicate that pyroptosis is also a form of inflammatory cell death (Lin et al., 2020). Pyroptosis has recently been associated with diabetic complications (Yang et al., 2019a; Zhang et al., 2020; Gu et al., 2019a), particularly diabetic cardiomyopathy (Kar et al., 2019; Li et al., 2014b; Yang et al., 2019b). However, evidence of pyroptosis in DKD is limited (Gu et al., 2019a; Wang et al., 2019). In the present study, we observed renal injuries in DKD rats accompanied by the overexpression of pyroptotic-related proteins (GSDMD, NLRP3, caspase-1, and IL-1β), which mainly localized to renal tubules. However, in TSF-treated DKD rats, renal injuries were alleviated, and pyroptotic-related proteins were downregulated, which suggests that pyroptosis of renal tubular epithelium occurs in DKD, and TSF imparts anti-pyroptotic effects.

To confirm our hypothesis, HK-2 cells stimulated by AGEs were used in an *in vitro* experiment. First, we intended to prove the existence of pyroptosis. We observed increased cell death in AGE-stimulated HK-2 cells by calcein-AM/PI staining. Furthermore, we used the caspase-1 inhibitor YVAD to pretreat cells before AGE stimulation, and the results showed that cell death significantly decreased, which suggests that cell death is mainly caspase-1-dependent. In addition, YVAD inhibited the expression of the pyroptotic executor GSDMD-N and cleaved-caspase-1, which were upregulated by AGE stimulation. These results support the hypothesis that pyroptosis occurred in AGE-stimulated HK-2 cells. Second, we found that TSF imparts an anti-pyroptotic effect as it reduced AGE-induced pyroptosis as well as the expression of GSDMD-N and cleaved-caspase-1. However, the mechanism of how TSF influences pyroptosis remains unclear.

Inflammasomes have been shown to trigger pyroptosis (Malik and Kanneganti 2017), which is accompanied by the production of cleaved caspase-1 (the active form of caspase 1). Among the known inflammasomes, the NLRP3 inflammasome is the most extensively studied. Accumulating evidence has shown that the inflammatory effects caused by the activation of the NLRP3 inflammasome play an important role in DKD (Shahzad et al., 2015; Qiu and Tang 2016; Wu et al., 2018). However, more evidence is still needed to determine whether the NLRP3 inflammasome triggers pyroptosis of renal resident cells in DKD. As previously reported (Hong et al., 2019), we also observed the activation of the NLRP3 inflammasome in AGE-stimulated HK-2 cells in this study, shown as a time-dependent increase of NLRP3, cleaved-caspase-1, and mature IL-1β expression, as well as IL-1β release. However, the activation of the NLRP3 inflammasome was inhibited by TSF in a dose-dependent manner. This result prompted us to further clarify whether TSF reduces pyroptosis by inhibiting the NLRP3 inflammasome. For this purpose, the exogenous agonist of NLRP3 inflammasome, nigericin, was used. Interestingly, the inhibitory effect of TSF on pyroptosis was abolished by nigericin. At the same time, the inhibited expression of molecules in the NLRP3-pyroptosis pathway was partially restored by nigericin. These results confirmed our hypothesis that TSF reduces pyroptosis by regulating the NLRP3 inflammasome.

Overproduction of ROS plays a pivotal role in the progression of DKD (Jha et al., 2016; Wei and Szeto 2019). ROS has also been reported to be one of the crucial elements of NLRP3 activation (Tschopp and Schroder 2010; Abais et al., 2015). TXNIP is a negative regulator of anti-oxidation and is involved in diabetes (Yoshihara et al., 2014). In physiological conditions, TRX binds to TXNIP and inhibits its activity. However, in some pathological conditions, excessive ROS promotes the dissociation of TRX and TXNIP (Lin et al., 2020). The detached TXNIP interacts with NLRP3 molecules and activates the NLRP3 inflammasome (Zhou et al., 2010; Han et al., 2018). In our previous study (published in Chinese), TSF was found to impart anti-oxidative effects on db/db mice. In the present study, we also found that TSF decreases ROS production and TXNIP expression in AGE-stimulated HK-2 cells. Importantly, colocalization of TXNIP-NLRP3 decreased with TSF treatment, which indicates that TSF affects TXNIP-NLRP3 interactions. These results suggest that ROS-TXNIP may be the target of TSF.

**FIGURE 7:**
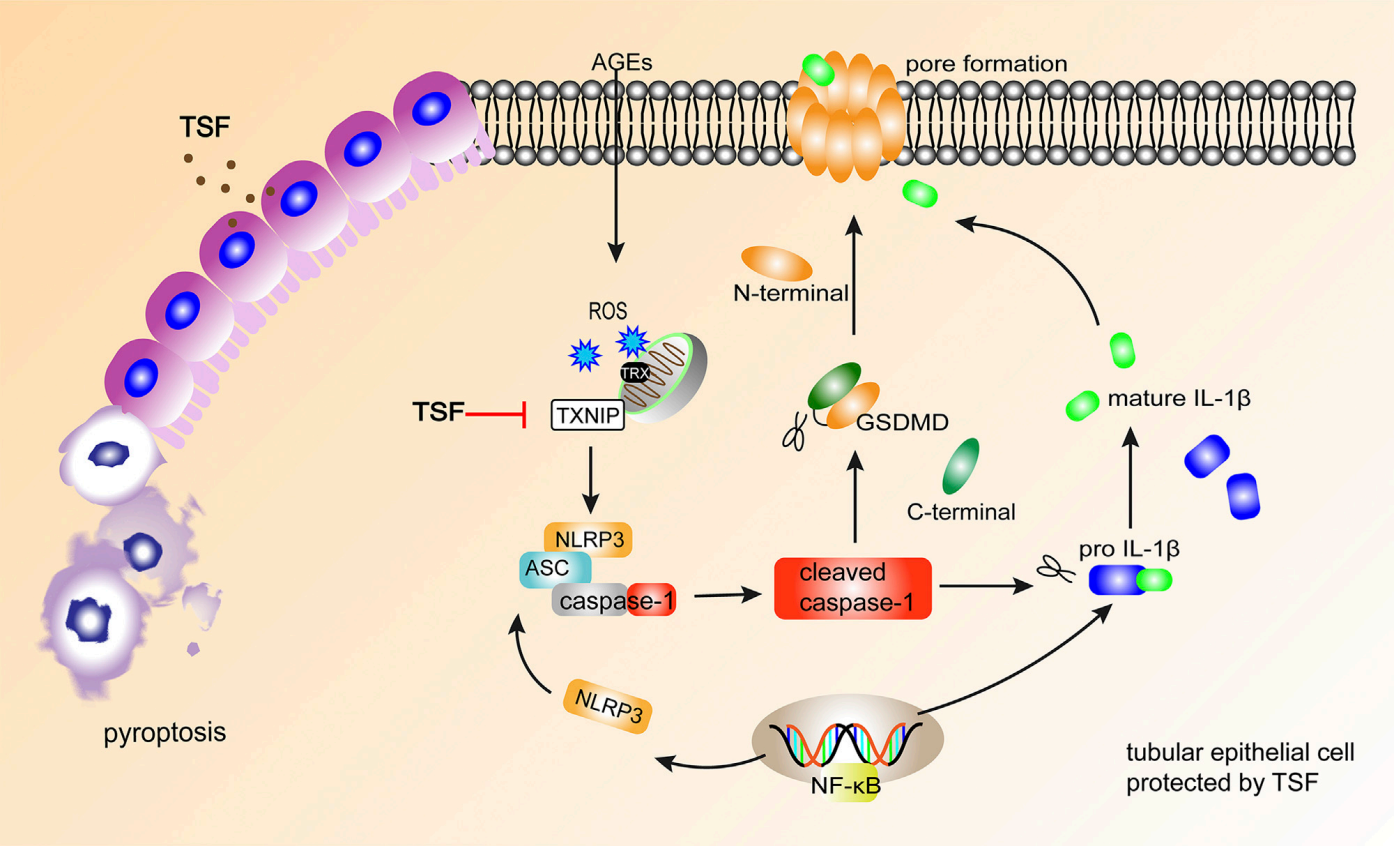
TSF alleviates tubular injury in DKD by imparting anti-pyroptotic effects *via* the TXNIP-NLRP3-GSDMD axis. In DKD, AGEs induce NLRP3-related pyroptosis in tubular epithelium, accompanied by IL-1β release. TSF protects tubular epithelium cells from pyroptosis by inhibiting the TXNIP-NLRP3-GSDMD pathway.

In conclusion, the present study demonstrated that TSF reduces the pyroptosis of tubular epithelia *via* the TXNIP-NLRP3-GSDMD axis (Figure 7). This might be an important mechanism of TSF in DKD therapy. However, limitations are present in this study. Some other inflammasomes, such as the pyrin inflammasome (Heilig and Broz 2018) and AIM2 inflammasome (Gao et al., 2019), have also been reported to initiate pyroptosis, but the present study only focused on NLRP3-related pyroptosis. Second, which components of TSF contribute to the anti-pyroptotic effect is not clear. In our next study, we will continue to explore the mechanism of pyroptosis in DKD as well as the effective components of TSF that regulate pyroptosis.

## Data Availability Statement

The original contributions presented in the study are included in the article/Supplementary Material, further inquiries can be directed to the corresponding authors.

## Ethics Statement

The animal study was reviewed and approved by China-Japan Friendship Hospital, Institute of Clinical Medical Science (No. 2012-A04).

## Author Contributions

PL and HZ designed the experiments. TZ, HZ, MY, XZ, and XD performed the animal experiments. YW, QW, and JL conducted the molecular biology experiments. NL, YC, and LP analyzed and interpreted the data. HZ and PL wrote the manuscript.

## Funding

The National Natural Science Foundation of China (Grant No. 81620108031) and the Natural Science Foundation of Beijing, China (Grant No. 7192191) supported this study.

## Conflict of Interest

The authors declare that the research was conducted in the absence of any commercial or financial relationships that could be construed as a potential conflict of interest.
