# Supplementary material for: Tangshen Formula Attenuates Diabetic Kidney Injury by Imparting Anti-pyroptotic Effects via the TXNIP-NLRP3-GSDMD Axis
Source: Front Pharmacol. 2021 Jan 29;11:623489. doi: 10.3389/fphar.2020.623489 (PMC7880163; doi:10.3389/fphar.2020.623489)
Supplement: Supplementary file 1 [file datasheet1.pdf]

Control

caspase-1 & GSDMD & DAPI

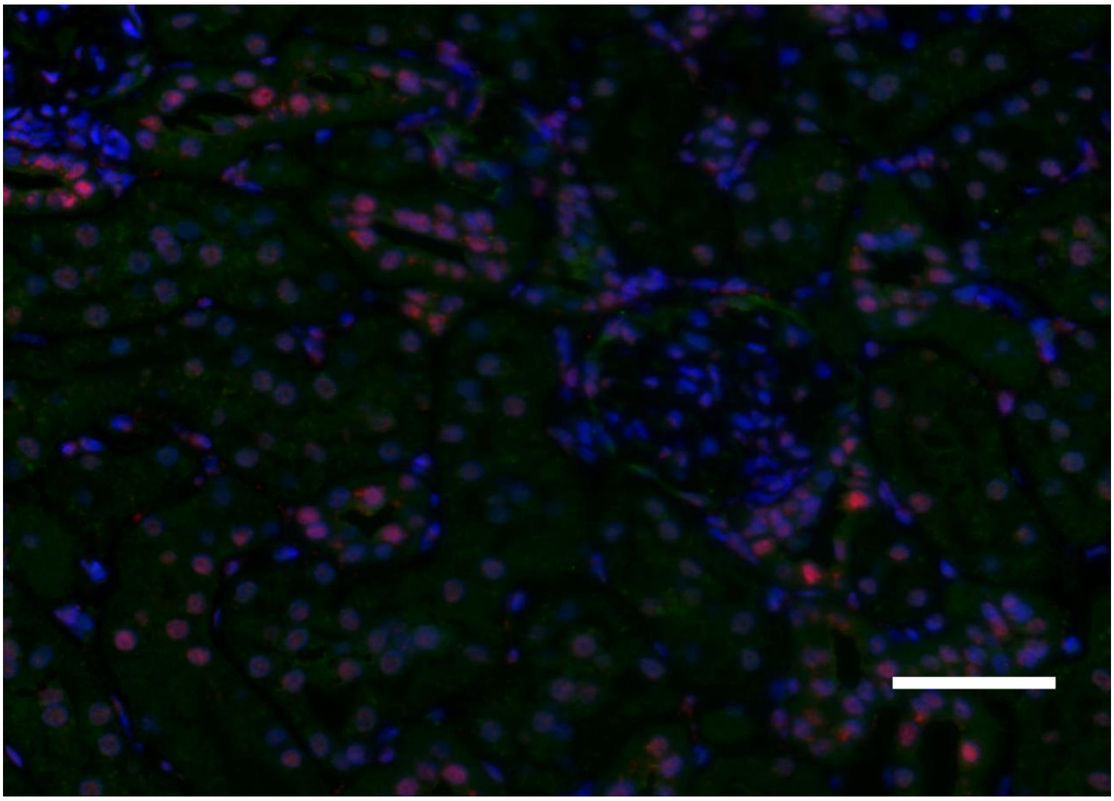

caspase-1 & NLRP3 & DAPI

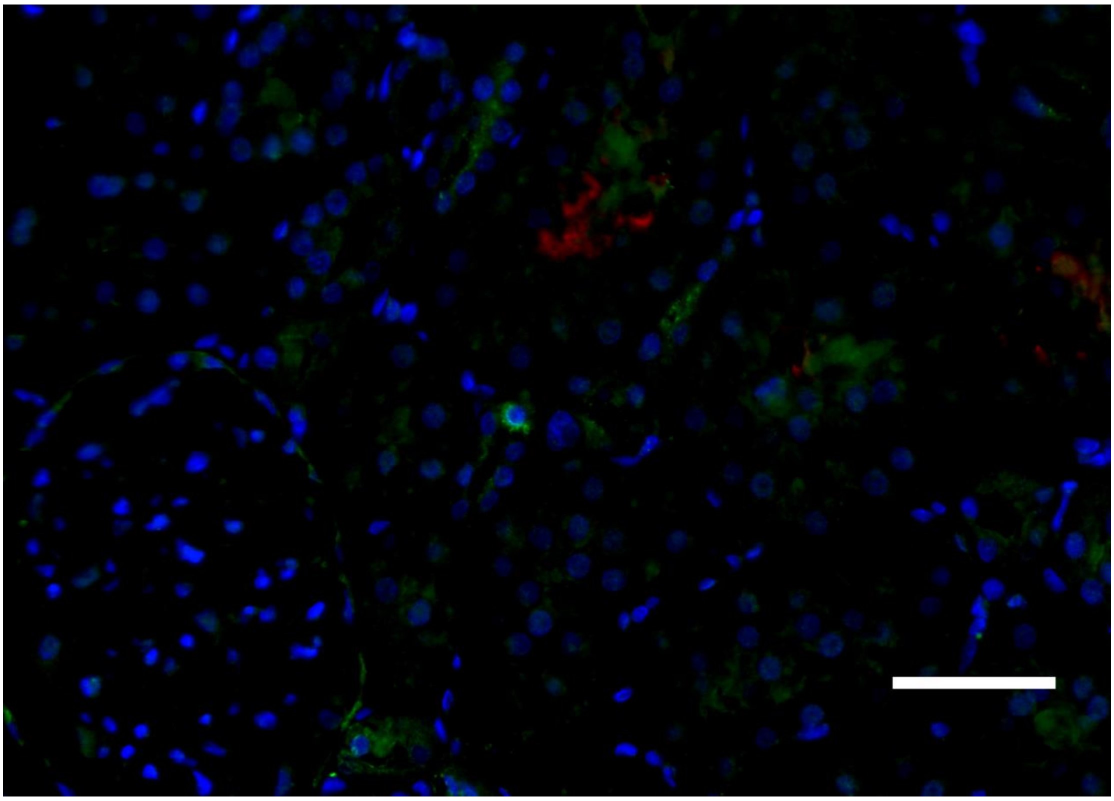

Model

caspase-1 & GSDMD & DAPI

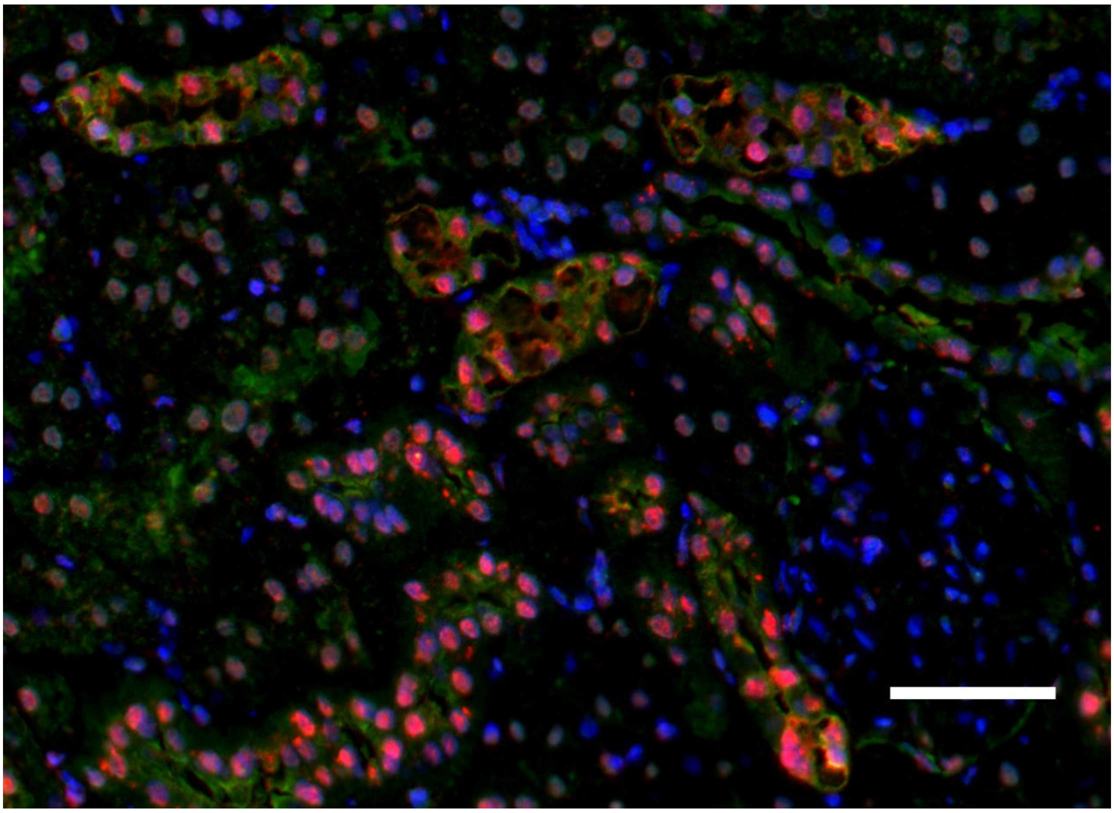

caspase-1 & NLRP3 & DAPI

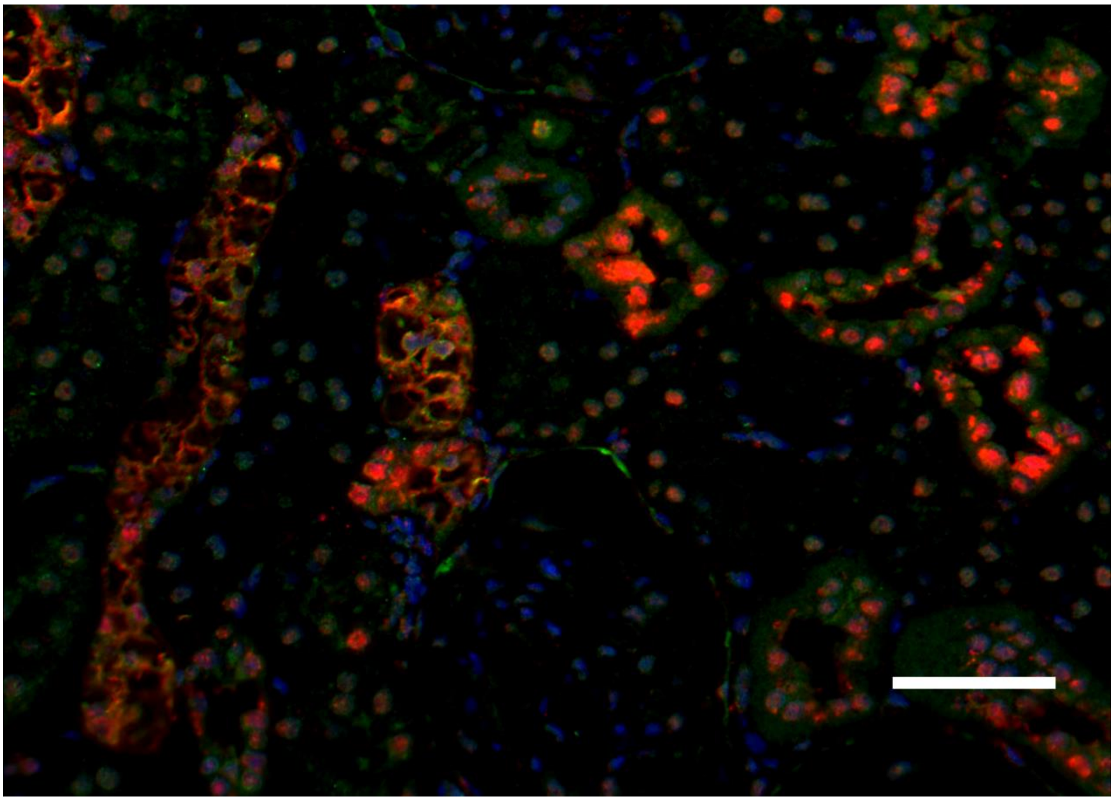

Model+TSF

caspase-1 & GSDMD & DAPI

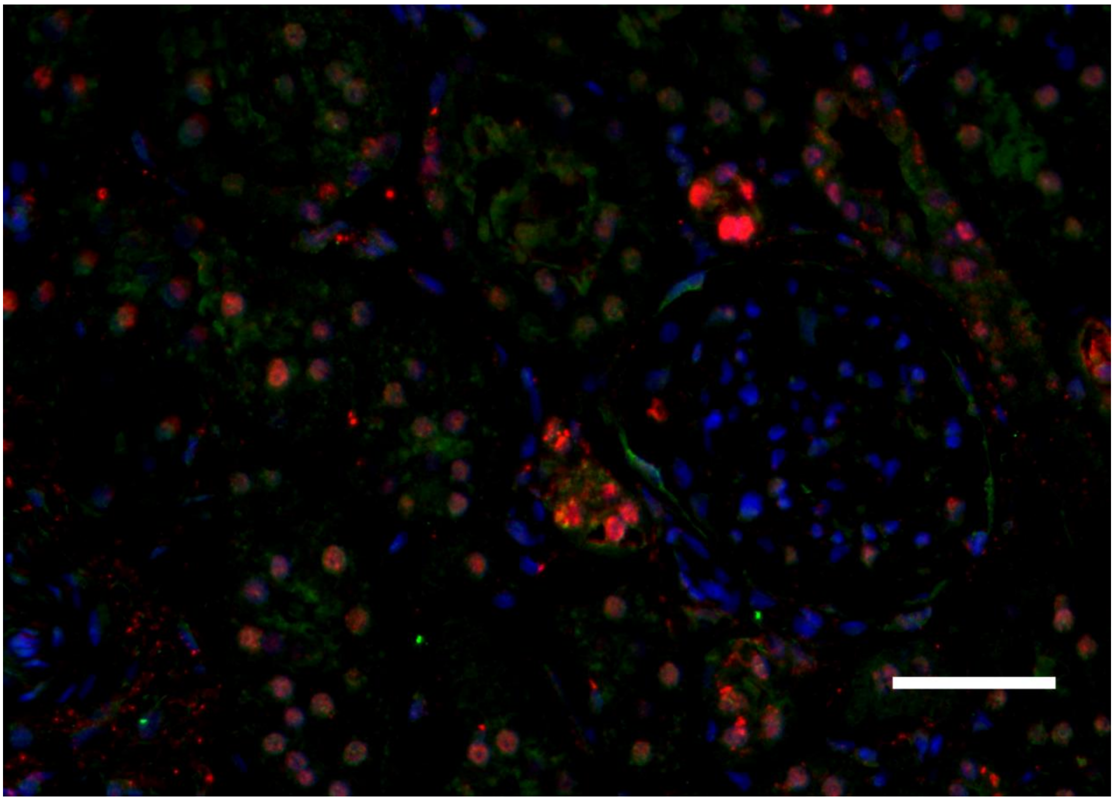

caspase-1 & NLRP3

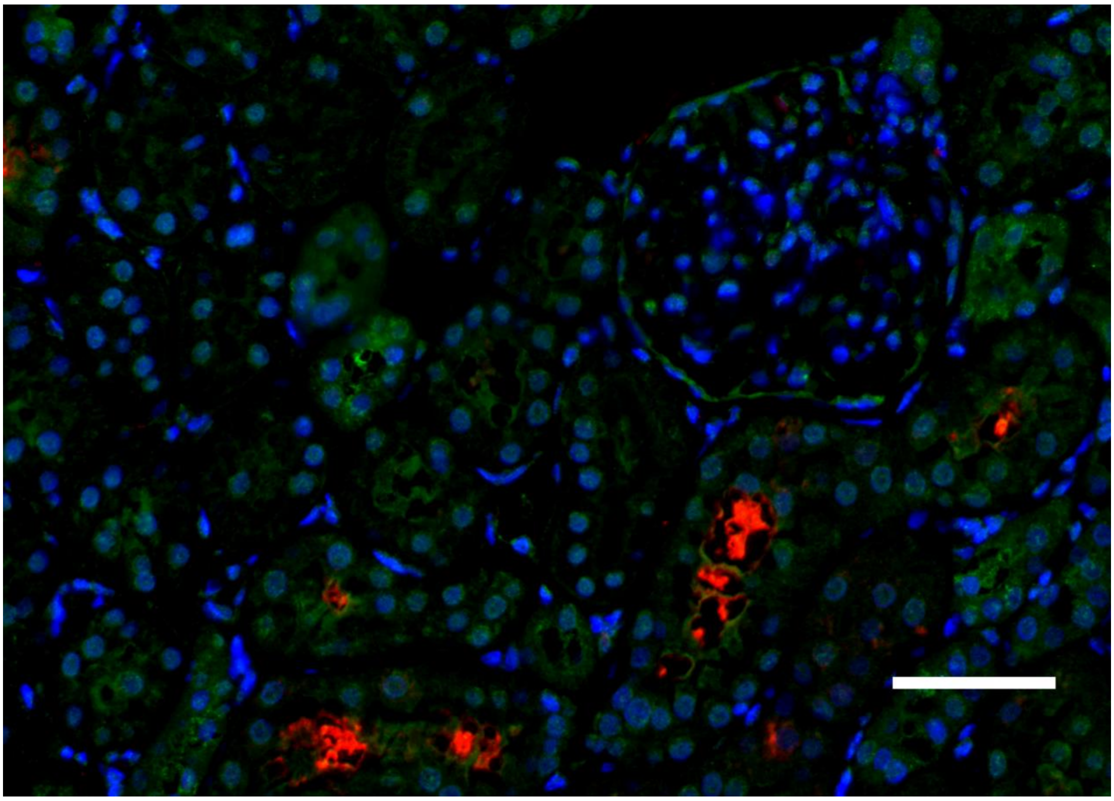

Supplement 2. Original pictures of Fig.2G. Scale bar=50μm
