# Supplementary figures and images for: Tangshen Formula Attenuates Diabetic Kidney Injury by Imparting Anti-pyroptotic Effects via the TXNIP-NLRP3-GSDMD Axis
Source: Front Pharmacol. 2021 Jan 29;11:623489. doi: 10.3389/fphar.2020.623489 (PMC7880163; doi:10.3389/fphar.2020.623489)

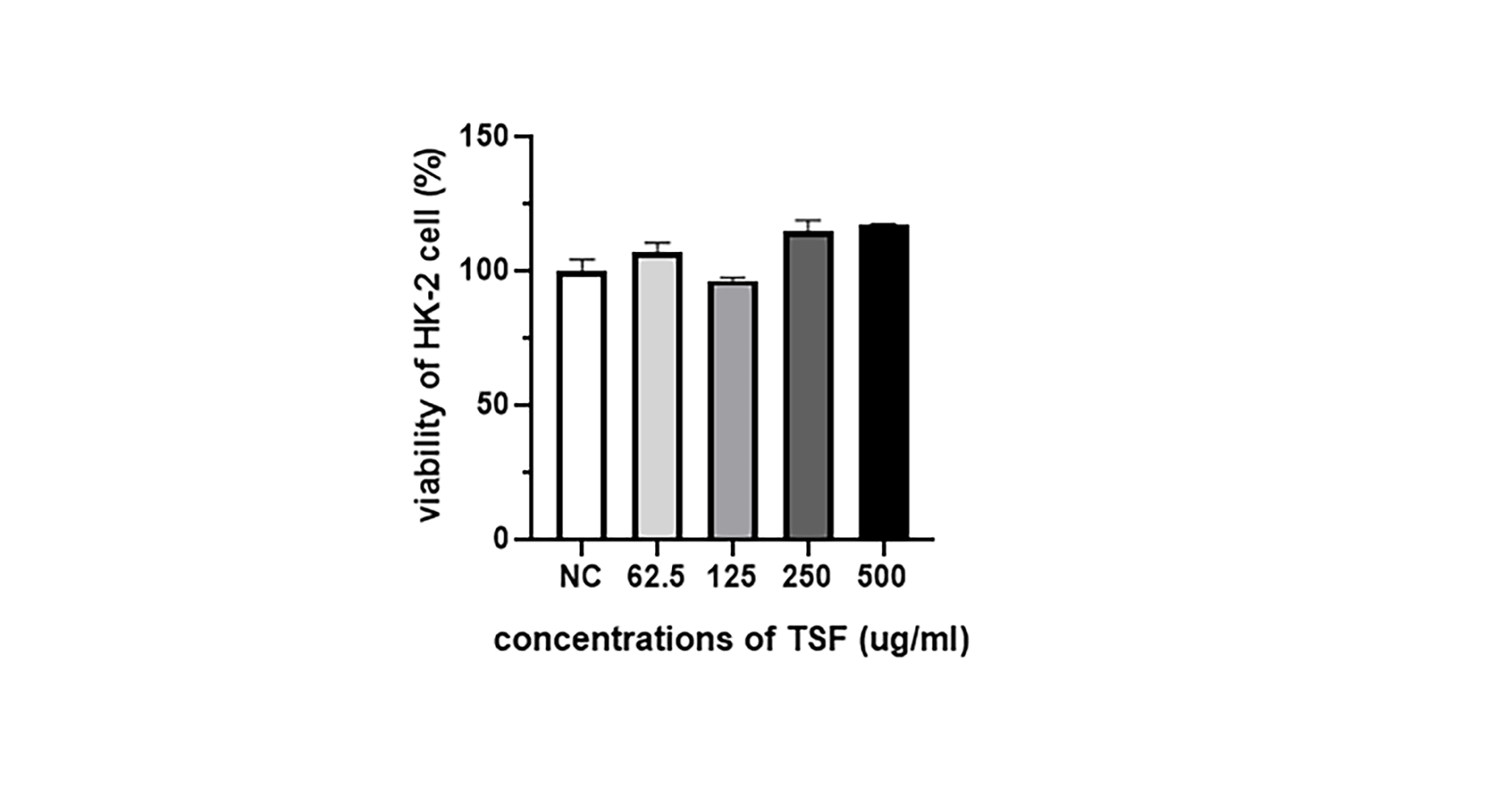

Supplement: Supplementary file 2 [file image1.tif]
